# Hearing loss and the COVID-19 pandemic

**DOI:** 10.1186/s13104-022-06120-1

**Published:** 2022-06-27

**Authors:** Yukiko Wagatsuma, Kaori Daimaru, Shiqi Deng, Jou-Yin Chen

**Affiliations:** 1 Department of Clinical Trials and Clinical Epidemiology, Faculty of Medicine, University of Tsukuba, 1-1-1 Tennodai, Tsukuba, Ibaraki 305-8575 Japan; 2 Department of Clinical Trials and Clinical Epidemiology, Graduate School of Comprehensive Human Sciences, University of Tsukuba, 1-1-1 Tennodai, Tsukuba, Ibaraki 305-8575 Japan

**Keywords:** Health examination, Hearing loss, COVID-19

## Abstract

**Objective:**

Hearing loss is an important public health problem. Its causes vary, including infections, noise, and aging. The first wave of the COVID-19 pandemic occurred in April 2020 in Japan. During the pandemic, people were urged to stay at home and drastically changed their lifestyles. This study aimed to examine hearing loss before and during the pandemic. The prevalence during the pandemic after April 2020 was compared for the period in 2019. Study subjects were those who received health checkups in both periods. Hearing loss was defined as a hearing threshold of > 30 dB at 1 kHz and > 40 dB at 4 kHz in either ear using pure-tone audiometry.

**Results:**

A total of 2367 persons presented in both 2019 and 2020. The overall rates of hearing loss were 9.5% and 13.2% before and after the pandemic, respectively. After controlling for age, sex, current smoking, regular exercise and alcohol consumption, the rate of hearing loss showed a significant increase in 2020 (p =  < 0.0001). With age stratification, an increase was observed in the participants aged < 40 years (1.3% vs. 3.1%, p < 0.001) and 40–59 years (7.2% vs. 12.6%, p < 0.001). Further studies are needed to confirm the impact of the COVID-19 pandemic on hearing loss.

**Supplementary Information:**

The online version contains supplementary material available at 10.1186/s13104-022-06120-1.

## Introduction

Hearing loss is a major public health issue. It is caused by infections, exposure to loud sounds, chronic diseases, and aging. Recently, the overuse of earphones has been associated with the development of hearing loss [1]. A worldwide study revealed that the prevalence of hearing loss varied among the 212 participating countries, ranging from < 11% to > 28% [2]. The COVID-19 pandemic, which began in 2020, dramatically changed people’s lifestyles.

There are reports of substantial lifestyle changes during the COVID-19 pandemic. The Government of Japan has been promoting remote work, and many workers changed to remote work during the emergency declaration [3]. A study from the U.S. reported that 72% of the general population changed to remote work, and remote workers had increases in on-working day sedentary behavior and stress, with a greater decline in physical functioning [4]. Lifestyle changes during the pandemic affected workers’ stress due to strict regulations to avoid infection [5]. A Japanese survey among workers revealed longer sedentary behaviors, such as sitting, for those who worked from home compared to those who worked at workplaces [3]. The frequency and duration of earphone and headphone use have been increasing as remote work increases. The association between hearing loss and personal listening devices has also been reported [6]. A large-scale cohort study showed that users listening to high sound levels increased their hearing thresholds [7].

There have been emerging publications on sudden sensorineural hearing loss after COVID-19 infection [8–12]. All these investigations were based on COVID-19 patients, and systematic reviews and meta-analyses were conducted to report the association between sudden sensorineural hearing loss and COVID-19 infection [13–17]. A meta-analysis showed that the rate of hearing loss was 3.1%, which was statistically significant in patients with COVID-19 [16]. A study involving young and middle-aged health care workers showed that mild and moderate COVID-19 disease did not permanently affect hearing function [18]. Further studies with follow-up assessments are needed. Regarding the use of face masks during the pandemic, sound attenuation and the inability to read lips were reported as the main concerns [19, 20].

The current study aimed to examine hearing loss before and during the COVID-19 pandemic by comparing the prevalence among participants in regional health checkup centers in Japan. Furthermore, this study aimed to clarify any subgroups whose hearing ability may have been affected, e.g., different age groups show different situations of hearing loss before and during the pandemic. Lifestyle-related information was also examined to determine its association with hearing loss.

## Main text

### Study population and study subjects

Persons who received health checkups conducted in a regional health care center in Mito, Japan, were invited to participate in the study. All persons with hearing ability measurements were included in the study. Health information for the pandemic period came from the period of April 2020–March 2021 (we denote as “2020”) and for the prepandemic period of April 2019–March 2020 (we denote as “2019”). Study subjects were those who received health checkups in both periods.

### Information collected and used in the current study

The information used for the analysis included the date of health examination, age, sex, body mass index (BMI), and medical history, which included medication use for hypertension, diabetes, and dyslipidemia, and history of stroke and heart disease. Self-reported information on current smoking, alcohol consumption, and regular exercise were also collected and analyzed. Regular exercise was defined as > 30 min/day and > 2 times/week based on a standardized questionnaire for lifestyle-related diseases by the Ministry of Health, Labour and Welfare, Japan.

### Measurement of hearing ability

Pure-tone audiometry was conducted by trained medical staff using an AA-57 or AA-58 audiometer (Rion Co, Ltd., Tokyo, Japan). Hearing loss was defined as a hearing threshold of > 30 dB at 1 kHz and > 40 dB at 4 kHz in either ear, according to the National Health Examination Guidelines (Industrial Safety and Health Act, Ordinance on Industrial Safety and Health, Articles 43, 44, 45).

### Statistical analysis

For the characteristics of the study participants, McNemar’s test and paired t test for continuous variables were used for categorical variables and continuous variables, respectively, to examine the differences between the two periods. The analysis was further conducted with the stratification of age groups (< 40, 40–59, 60 + years), and the differences in the prevalence between the two periods were examined. Lifestyle changes were examined for current smoking, alcohol consumption, and regular exercise and compared between the two periods using McNemar’s test. To compare the rate of hearing loss between the periods, a generalized estimating equation (GEE) with repeated measures by examination year was used, and showed parameter estimates with 95% confidence intervals (95% CIs). The final model included variables of examination period (2020 vs. 2019), age (continuous), sex, current smoking, alcohol consumption, and regular exercise. Statistical analyses were performed using IBM SPSS Statistics version 27. Statistical significance was defined as a two-sided p value < 0.05.

## Results

Table 1 shows the characteristics of the study subjects in 2019 and 2020. The number of study subjects was 2367. The mean age in 2019 was 46.4 years (SD 13.8, range 18–86 years). Males accounted for 58.8% of the sample. Current medication use for hypertension and dyslipidemia and a medical history of stroke were significantly different between the two periods (Table 1). The prevalence of hearing loss in 2020 was significantly higher than that in 2019 (9.5% (225/2367) and 13.2% (313/2367), respectively; p < 0.0001).

**Table 1:** Characteristics of study subjects

	2019	2020	p value^e^
Sex, male, %	58.8% (1391/2367)		
Age, years, mean (SD)	46.4 (13.8)	47.4 (13.8)	< .0001
BMI, kg/m^2^, mean (SD)	23.5 (4.0)	23.7 (4.1)	< .0001
Hearing loss, %	9.5% (225/2367)	13.2% (313/2367)	< .0001
Medication, %			
Hypertension^a^	15.8% (353/2232)	17.7% (394/2232)	< .0001
Diabetes^b^	4.7% (106/2236)	5.2% (117/2236)	0.071
Dyslipidemia^c^	11.4% (255/2235)	12.7% (284/2235)	0.0003
Medical history, %			
Stroke^c^	1.7% (37/2235)	2.0% (45/2235)	0.039
Heart disease^d^	2.5% (56/2234)	2.6% (59/2234)	0.701

^a^Missing n = 135, ^b^Missing n = 131, ^c^Missing n = 132, ^d^Missing n = 133, ^e^P value was calculated using paired t-test for continuous variables and McNemar's test for categorical variables

**Fig. 1:**
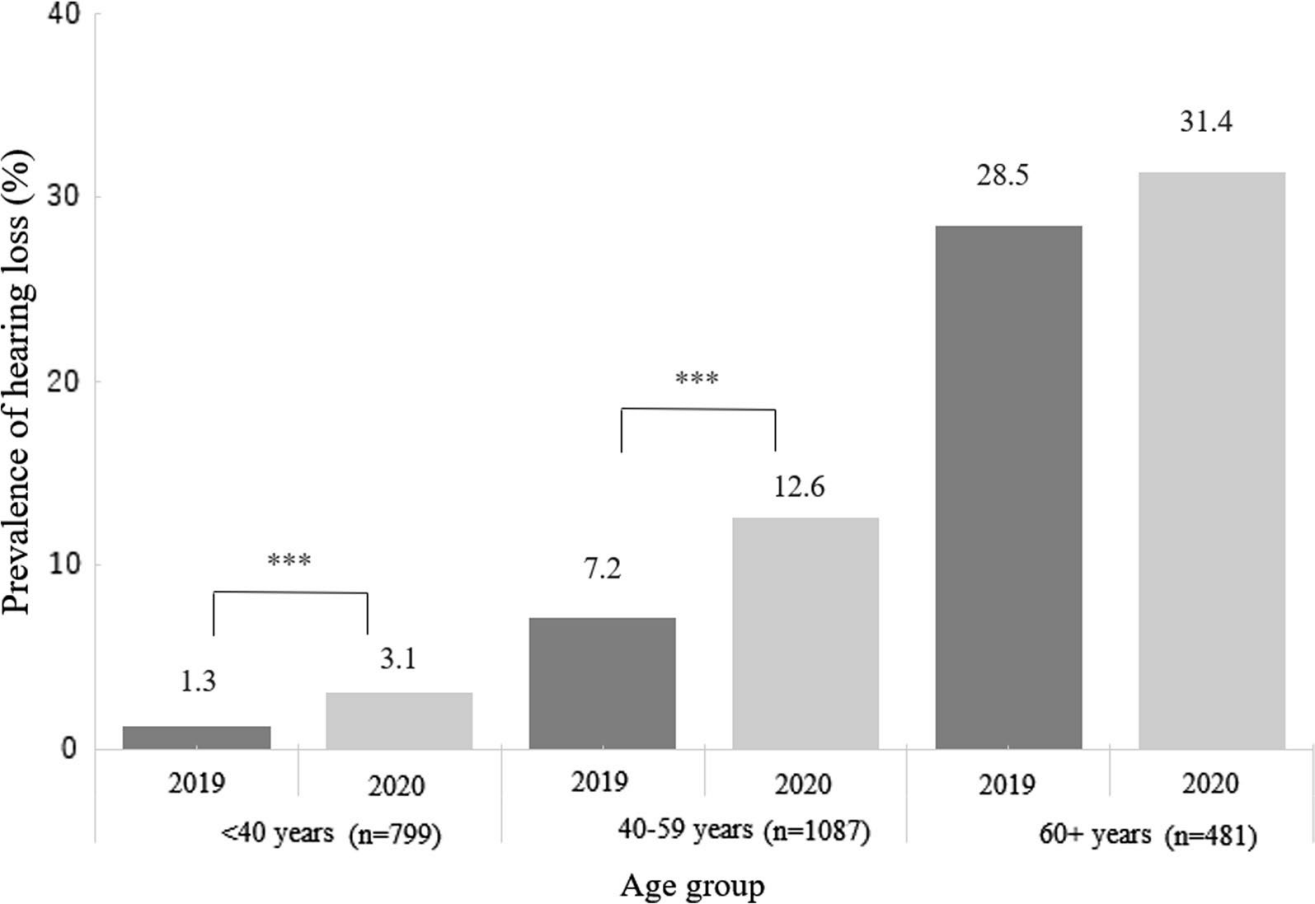
Prevalence of hearing loss in 2019 and 2020, n = 2367. P value was calculated using McNemar's test. Asterisks (*) denote a significant difference (***p < 0.001) in the prevalence of hearing loss in 2020 compared to 2019 by age group.

By age stratification analysis, the rates of hearing loss in 2020 for participants aged under 40 years (1.3% (10/799) vs. 3.1% (25/799), respectively, p < 0.001) and participants aged 40 to 59 years (7.2% (78/1087) vs. 12.6% (137/1087), respectively, p < 0.001) were significantly higher than those in 2019 (Fig. 1).

Current smoking decreased in 2020 (23.4% (523/2234) in 2019 vs. 22.4% (501/2234) in 2020, p = 0.032) and there were no significant differences in regular exercise and alcohol consumption between the two periods (Additional file 1: Table S1). With the stratification of age groups, current smoking only showed a significant difference between the years for the group of participants aged 60 years and older (16.5% (74/449) vs. 14.5% (65/449), p = 0.035).

In a GEE univariable analysis, the period of the COVID-19 pandemic in 2020 showed a significantly higher rate of hearing loss (beta estimate [95% CI] = 0.372 [0.260–0.484], p < 0.0001; Additional file 1: Table S2). Males had a higher rate of hearing loss (beta estimate [95%CI] = 0.668 [0.389–0.947], p < 0.0001). Participants with regular exercise and alcohol consumption showed a significant increase in hearing loss in 2020 (beta estimate [95% CI] = 0.310 [0.102–0.517], p = 0.003 and 0.540 [0.311–0.768], p < 0.0001, respectively).

The final GEE multivariable model with the variables of age, sex, regular exercise, current smoking, and alcohol consumption, a significantly higher rate of hearing loss was observed during the pandemic period (beta estimate [95% CI] = 0.339 [0.208–0.471], adjusted odds ratio (aOR) = 1.404, p < 0.0001). Male sex was associated with a higher rate of hearing loss (beta estimate [95% CI] = 0.668 [0.389–0.947], p < 0.0001, aOR = 1.950) (Table 2). The effect modification of the variables included in the model was examined, and the interactions were assessed. There were no significant interactions.

**Table 2:** Generalized Estimating Equations (GEE) with repeated measures of hearing loss, n = 2345 (multivariable analysis)

Variable	Beta estimate	Standard Error	95% CI	p value	aOR
Health checkup year					
2020	0.339	0.067	0.208–0.471	< .0001	1.404
2019 (reference)					
Sex					
Male	0.668	0.142	0.389–0.947	< .0001	1.950
Female (reference)					
Smoking					
Yes	0.223	0.137	−0.045–0.492	0.1032	1.250
No (reference)					
Regular exercise					
Yes	0.027	0.116	−0.201–0.254	0.8173	1.027
No (reference)					
Alcohol consumption					
Yes	0.124	0.123	−0.116–0.364	0.3118	1.132
No (reference)					

Hearing loss was defined as a hearing threshold of > 30 dB at 1 kHz and > 40 dB at 4 kHz in either ear with pure-tone audiometry

OR and p value were calculated using Generalized Estimating Equations Model

OR was also adjusted for age as a continuous variable

aOR: adjusted odds ratio, 95% CI: 95% confidence interval

## Discussion

This study showed that the prevalence of hearing loss among people < 60 years of age significantly increased during the COVID-19 pandemic compared to the prepandemic period. Surprisingly, people under 40 years of age were also significantly affected. This is the first study to report an association between hearing loss and the COVID-19 pandemic in the general population.

This study used a definition of hearing loss with a hearing threshold of > 30 dB at 1 kHz and > 40 dB at 4 kHz in either ear. This was adopted according to national health examination guidelines in Japan. The World Health Organization recently published a report on hearing loss, which was defined as “a *person is said to have hearing loss if their hearing capacity is reduced, and they are not able to hear as well as someone with normal hearing. “Normal” hearing typically refers to hearing thresholds of 20 dB or better in both ears”* [1]. According to the grading system stated in this report, the thresholds used in the current study were those for moderate hearing loss. A systematic review showed that mild COVID-19 disease causes audio-vestibular damage [16]. There would be an increase in the number of cases if milder cases were included. The standard health examination procedure in this study followed the national guideline definition, and health checkup records frequently lack a threshold level, that is, hearing loss is reported as yes or no. Therefore, sensitivity analysis could not be conducted. This limits the comparability with other international studies. Although age-related hearing loss progresses slowly, it would be possible that some study subjects were in the early stages of “hearing loss”, and they may have progressed after a year.

Systematic reviews have described possible neuroinvasive actions for hearing loss with SARS-CoV-2 infection [14, 16, 17]. The current study lacked information on infection. We cannot deny the influence of the direct action of the virus, and it is uncertain whether asymptomatic infections also cause hearing loss. According to the periodical updates on COVID-19 by the National Institute of Infectious Diseases, the number of new infections had continued to increase, with infections just below 90 per 100,000 persons in the Tokyo metropolitan area at the beginning of 2021 [21].

Rather, this might be affected by other factors such as lifestyle changes, for example, the long-term use of earphones for online communication during the pandemic in 2020. Many people changed to remote work and stayed home for longer periods [3, 4]. Personal communication and entertainment devices with earphone or headphone use have increased recently [1], and it would have been further facilitated by the COVID-19 pandemic. Although the study did not include information on earphone use, the study subjects in the current study might have been exposed to earphone use for a longer duration. Further information is required to monitor inappropriate earphone use.

Tobacco smoking has been reported to influence hearing ability [13, 22]. This study did not show this effect on hearing loss. Fortunately, remote work shifts and stay-at-home policies seem to influence smoking behavior. This study showed that the prevalence of current smoking decreased during the pandemic. A study conducted in Italy on lifestyle changes during the COVID-19 pandemic also showed a decreasing trend in smoking [24].

Physical exercise and alcohol consumption have been shown to be associated with hearing loss [25–27]. However, this study found that there was no significant association when adjusting for age, sex, and smoking. In this study, the prevalence of regular exercise and alcohol consumption did not change after the pandemic. The study used the definition of regular exercise as “ > 30 min/day and > 2 times/week.” This may not have been sensitive enough to capture the association with hearing loss. In addition, self-reported information with simple yes or no options may limit the conclusion due to these factors, e.g., no dose–response relationship was assessed.

## Limitations

This study could not assess causality between hearing loss and the COVID-19 pandemic due to its cross-sectional design. In addition, the health checkup data used in this study did not include information on SARS-CoV-2 infection status, ontological history or inappropriate earphone use. Further studies are required to confirm the direct and indirect impacts of the COVID-19 pandemic on hearing loss.

## Supplementary Information

**Additional file 1: Table S1.** Lifestyle changes between 2019 and 2020. **Table S2.** Generalized estimating equations (GEE) with repeated measures of hearing loss, n=2345 (univariable analysis).

## Data Availability

The data used and/or analyzed during the study are available from corresponding author on reasonable request.
