# Supplementary material for: Hearing loss and the COVID-19 pandemic
Source: BMC Res Notes. 2022 Jun 27;15:228. doi: 10.1186/s13104-022-06120-1 (PMC9235250; doi:10.1186/s13104-022-06120-1)
Supplement: Supplementary file 1 — Additional file 1: Table S1. Lifestyle changes between 2019 and 2020. Table S2. Generalized estimating equations (GEE) with repeated measures of hearing loss, n=2345 (univariable analysis). [file 13104_2022_6120_MOESM1_ESM.docx]

| Additional table 1.　Lifestyle changes between 2019 and 2020 | | |  |  |
| --- | --- | --- | --- | --- |
| Lifestyle variable | n | 2019 | 2020 | p value^d)^ |
| Current smoking^a)^, yes |  |  |  |  |
| All ages | 2234 | 23.4% (523/2234) | 22.4% (501/2234) | 0.032 |
| <40 | 702 | 24.9% (175/702) | 23.4% (164/702) | 0.144 |
| 40-59 | 1083 | 25.3% (274/1083) | 25.1% (272/1083) | 0.864 |
| 60+ | 449 | 16.5% (74/449) | 14.5% (65/449) | 0.035 |
| Regular exercise^b)^, yes |  |  |  |  |
| All ages | 2235 | 20.9% (467/2235) | 21.1% (471/2235) | 0.866 |
| <40 | 702 | 19.1% (134/702) | 19.7% (138/702) | 0.775 |
| 40-59 | 1083 | 17.7% (192/1083) | 18.3% (198/1083) | 0.654 |
| 60+ | 450 | 31.3% (141/450) | 30.0% (135/450) | 0.576 |
| Alcohol consumption^c)^, yes |  |  |  |  |
| All ages | 2229 | 21.1% (471/2229) | 21.9% (487/2229) | 0.217 |
| <40 | 700 | 10.7% (75/700) | 10.7% (75/700) | 1.000 |
| 40-59 | 1079 | 24.5% (264/1079) | 25.1% (271/1079) | 0.470 |
| 60+ | 450 | 29.3% (132/450) | 31.3% (141/450) | 0.211 |

Hearing loss was defined as a hearing threshold of >30 dB at 1 kHz and > 40 dB at 4 kHz in either ear with
pure-tone audiometry.

a) Missing n=133, b) > 30 min/day, ​> 2 times/week, missing n=132, c) Defined as drinking every day, missing n=138

d) P value was calculated using the McNemar's test.

Additional table 2. Generalized estimating equations (GEE) with repeated measures of hearing loss, n=2345 (univariable analysis)

| Variable | Beta estimate | Standard Error | 95% CI | p value | OR |
| --- | --- | --- | --- | --- | --- |
| Health checkup year |  |  |  |  |  |
| 2020 | 0.372 | 0.057 | 0.260-0.484 | <.0001 | 1.451 |
| 2019 (reference) |  |  |  |  |  |
| Sex |  |  |  |  |  |
| Male | 0.556 | 0.125 | 0.311-0.801 | <0.001 | 1.744 |
| Female (reference) |  |  |  |  |  |
| Smoking^a)^ |  |  |  |  |  |
| Yes | 0.134 | 0.119 | -0.010-0.367 | 0.262 | 1.143 |
| No (reference) |  |  |  |  |  |
| Regular exercise^b)^ |  |  |  |  |  |
| Yes | 0.310 | 0.106 | 0.102-0.517 | 0.003 | 1.363 |
| No (reference) |  |  |  |  |  |
| Alcohol consumption^c)^ |  |  |  |  |  |
| Yes | 0.540 | 0.117 | 0.311-0.768 | <.0001 | 1.715 |
| No (reference) |  |  |  |  |  |

a) Missing n=154; b) Missing n=153; c) Missing n=160

Abbreviations: OR, odds ratio; 95% CI, 95% confidence interval

Hearing loss was defined as a hearing threshold of >30 dB at 1 kHz and > 40 dB at 4 kHz in either ear with pure-tone audiometry.

OR and p value were calculated using Generalized Estimating Equations Model
